# Post-endoscopy gastric cancer in Latin America: a multicentre cohort study from Colombia

**DOI:** 10.3389/fgstr.2026.1823329

**Published:** 2026-07-08

**Authors:** Hernando Marulanda-Fernandez, Juan Sebastián Frías Ordoñez, Gilberto Jaramillo Trujillo, Julián Parga Bermúdez, Jean Sebastián Barrero Gómez, Silvia Valentina Monsalve Rico, Elder Otero Ramos, Lina Otero Parra, William Otero-Regino

**Affiliations:** 1 Division of Gastroenterology, Universidad Nacional de Colombia, Bogotá, Colombia; 2 Division of Gastroenterology and Digestive Endoscopy, Hospital Central de la Policía, Bogotá, Colombia; 3 Division of Gastroenterology and Digestive Endoscopy, Hospital Occidente de Kennedy, Bogotá, Colombia; 4 Division of Gastroenterology and Digestive Endoscopy, Hospital Santa Clara, Bogotá, Colombia; 5 Department of Gastroenterology, Center for Gastroenterology and Endoscopy, Bogotá, Colombia; 6 Division of Gastroenterology, Hospital Internacional de Colombia, Bucaramanga, Colombia; 7 Research Group in Medical and Surgical Specialties, Fundación Cardiovascular de Colombia, Floridablanca, Colombia; 8 Division of Gastroenterology, Hospital San Rafael de Facatativá, Cundinamarca, Colombia; 9 Division of Gastroenterology, Hospital El Tunal, Bogotá, Colombia

**Keywords:** early detection, endoscopic quality, gastric cancer, *Helicobacter pylori*, Latin America, post-endoscopy gastric cancer

## Abstract

**Background:**

Post-endoscopy gastric cancer (PEGC), defined as gastric cancer diagnosed after a negative upper gastrointestinal endoscopy, is considered a key indicator of endoscopic quality. Although international studies estimate that PEGC accounts for 5–11% of gastric cancer cases, evidence from Latin America remains scarce.

**Methods:**

We conducted a retrospective multicentre observational study across three tertiary hospitals in Bogotá, Colombia (2024–2025). Adult patients with histologically confirmed gastric neoplasms were included. PEGC was defined as cancer diagnosed between 6 and 36 months after a negative endoscopy. Clinical characteristics, tumour location, and hospital outcomes were compared between PEGC and non-PEGC cases.

**Results:**

Amongst 599 patients, 71 met criteria for PEGC, corresponding to a frequency of 11.9% (95% CI: 9.5–14.7). Helicobacter pylori positivity was 67.1% overall and 70.4% amongst PEGC cases. PEGC was associated with higher rates of palliative management (23.9% vs 2.8%; OR 10.77; 95% CI: 5.09–22.77; p<0.001) and increased in-hospital mortality (16.9% vs 7.0%; OR 2.70; 95% CI: 1.33–5.46; p=0.004).

**Conclusion:**

PEGC was frequent in this Colombian cohort and associated with adverse hospital outcomes, highlighting missed diagnostic opportunities and the need to strengthen endoscopic quality standards in high-burden regions. These findings support the incorporation of PEGC rate as a quality indicator for upper gastrointestinal endoscopy in high-incidence regions.

## Introduction

Gastric cancer remains one of the foremost oncological challenges worldwide. In 2021, more than one million new cases and approximately 950,000 deaths were recorded globally, positioning it amongst the leading causes of cancer-related mortality (1–3). Although age-standardised incidence rates have declined progressively in several regions, the absolute number of cases continues to rise as a consequence of population ageing, with an estimated burden exceeding 22 million disability-adjusted life years attributable to this disease (1, 3, 4). This epidemiological paradox — a relative decline coupled with a persistently high absolute burden — underscores the need for more effective diagnostic strategies, particularly in high-incidence regions.

The geographical distribution of gastric cancer is markedly heterogeneous. Eastern Asia and Eastern Europe report the highest incidence rates; however, Latin America continues to rank amongst the regions with substantial disease burden, largely associated with a high prevalence of *Helicobacter pylori* infection, salt-rich dietary patterns, and sustained exposure to adverse socio-environmental conditions (1, 2, 5). In Colombia, gastric cancer remains one of the leading causes of cancer mortality, with a less pronounced decline in incidence and a disproportionate impact on rural populations and individuals with limited access to specialised healthcare services (2, 4). Within this context, late diagnosis remains the norm rather than the exception, restricting opportunities for curative treatment and contributing to adverse clinical outcomes.

Post-endoscopy gastric cancer (PEGC), also referred to as post-endoscopy upper gastrointestinal cancer (PEUGIC), is defined as gastric cancer diagnosed between 6 and 36 months following an upper gastrointestinal endoscopy deemed negative for malignancy (6, 7). This entity has emerged as a critical indicator of endoscopic quality, as it reflects lesions that were missed, mischaracterised, or interpreted as benign during the initial examination. International studies estimate that PEGC accounts for approximately 5–10% of gastric cancer cases, reaching figures close to 11% in multicentre analyses and systematic reviews (6, 7). Beyond its frequency, PEGC is clinically significant owing to its consistent association with diagnosis at more advanced stages, reduced likelihood of curative resection, and poorer overall prognosis (7).

Available evidence suggests that PEGC is a heterogeneous and multifactorial entity. Although many cases are believed to result from lesions that were present but not recognised during a previous examination, alternative mechanisms have also been described, including incomplete mucosal visualisation, inadequate biopsy sampling, failure to appropriately surveil premalignant gastric conditions, and, less frequently, rapidly progressive tumour biology (6–8). Several patient-related factors, such as Helicobacter pylori infection, gastric atrophy, intestinal metaplasia, advanced age, and diffuse-type histology, have been associated with an increased risk of PEGC, whereas lesion-related characteristics including small size, flat morphology, subtle mucosal appearance, and anatomically challenging locations may further hinder endoscopic detection (6–10). Importantly, a negative endoscopic examination should not be interpreted as complete protection against future gastric cancer development, as a minority of cancers may arise despite an apparently normal prior evaluation. Furthermore, advances in endoscopic quality assurance and image-enhanced endoscopy have improved the detection of subtle neoplastic and premalignant lesions, highlighting the potential role of systematic examination protocols, enhanced imaging technologies, and structured surveillance strategies in reducing the burden of PEGC (7, 8, 10).

Most of the current knowledge regarding post-endoscopy gastric cancer derives from Asia and Europe, regions where high disease incidence and the implementation of organised endoscopic screening programmes have enabled the development of large cohorts and systematic evaluations of endoscopic quality and oncological outcomes (6, 7, 9–11). In contrast, despite its considerable gastric cancer burden, Latin America remains underrepresented in the literature. The available evidence is limited, fragmented, and predominantly single-centre, with no multicentre studies comprehensively assessing the frequency, clinical and endoscopic characteristics, and associated outcomes of PEGC (12, 13). This knowledge gap reflects structural constraints, lack of standardised registries, and marked heterogeneity in endoscopic practice, leading to systematic underrepresentation of the region in international research.

Against this background, it is particularly relevant to determine whether the frequency and clinical impact of PEGC in Latin America mirror those reported in regions with structured screening programmes, or whether they instead reflect deficiencies in endoscopic quality rather than limitations in access to care. Accordingly, the present study aimed to characterise the frequency, clinical and endoscopic features, and hospital outcomes of post-endoscopy gastric cancer in a multicentre cohort of 599 patients treated at tertiary university referral hospitals in Colombia. We hypothesised that PEGC occurs at a frequency comparable to that observed in Asia and Europe but is associated with poorer clinical outcomes in a setting lacking organised screening programmes, thereby suggesting the possibility of modifiable factors related to endoscopic practice and underscoring the need for systematic interventions aimed at early detection.

## Materials and methods

### Study design and setting

We conducted a retrospective, multicentre observational cohort study across three tertiary university hospitals in Bogotá, Colombia, between January 2024 and October 2025. These centres were selected owing to their status as national referral institutions in gastroenterology, digestive oncology and oncological surgery, as well as the availability of fully digitised clinical, endoscopic and pathological records.

The exposure of interest was post-endoscopy gastric cancer (PEGC), defined as a histopathologically confirmed gastric neoplasm diagnosed between 6 and 36 months following a prior upper gastrointestinal endoscopy (oesophagogastroduodenoscopy, OGD) that was considered negative for malignancy. Eligible neoplasms included gastric adenocarcinoma, lymphoma, gastrointestinal stromal tumours, and neuroendocrine neoplasms. The comparator group comprised patients with no documented OGD within the 36 months preceding diagnosis.

A target sample size of 600 consecutive participants was prespecified based on an expected PEGC proportion of 8–10%, allowing identification of approximately 50–60 cases. This provided ≥80% statistical power for estimation and between-group comparisons, as well as for multivariable modelling with a minimum of five to six outcome events per covariate. The study adhered to the Strengthening the Reporting of Observational Studies in Epidemiology (STROBE) guidelines (14).

### Study population

**Figure 1:**
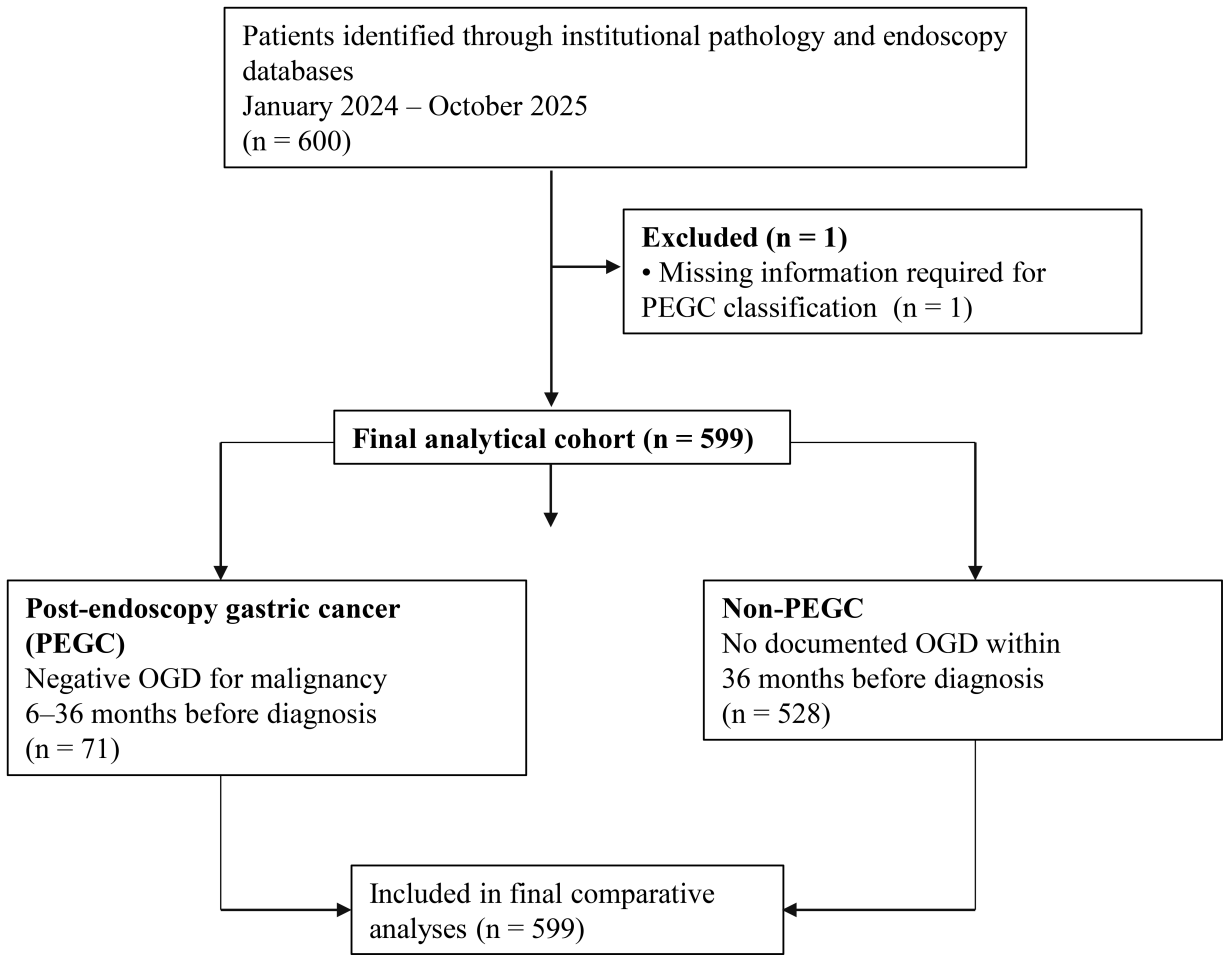
STROBE flow diagram of patient selection and exposure classification. A total of 600 potentially eligible patients were identified through institutional databases. After exclusion of one patient because of insufficient information for definitive exposure classification, 599 patients with histologically confirmed gastric neoplasms were included in the final analysis. Patients were classified as post-endoscopy gastric cancer (PEGC) when a negative upper gastrointestinal endoscopy had been documented 6–36 months before diagnosis, or as non-PEGC when no documented endoscopy was available within the preceding 36 months.

A total of 600 consecutive potentially eligible patients were initially identified through institutional pathology and endoscopy databases. One patient was excluded because the available records did not allow definitive exposure classification according to the predefined study criteria. Consequently, 599 patients were included in the final analysis, of whom 71 fulfilled criteria for PEGC and 528 were classified as non-PEGC. A study flow diagram is provided in **Figure 1**.

Adult patients (≥18 years) with histologically confirmed gastric neoplasms — including adenocarcinoma, lymphoma, gastrointestinal stromal tumours (GIST), and neuroendocrine tumours — diagnosed during the study period at participating institutions were eligible for inclusion. Verifiable endoscopic and pathological records were required to enable exposure classification.

Patients were excluded if their prior OGD had been performed at external institutions without verifiable documentation, if they had Siewert type I oesophageal adenocarcinoma, or if they had duodenal neoplasms, including those infiltrating the pyloric region.

### Exposure definition and comparison groups

Participants were categorised into two exposure groups. The PEGC group comprised patients diagnosed with gastric neoplasia between 6 and 36 months following an OGD reported as negative for neoplasia. The comparator group included patients diagnosed with gastric neoplasia without any documented OGD in the 36 months prior to diagnosis.

### Variables and outcomes

The primary exposure variable was the presence of PEGC (yes/no), operationalised according to the predefined 6–36 month interval.

The study aimed to compare clinical characteristics, anatomical distribution, and hospital outcomes between PEGC and non-PEGC cases.

Secondary outcomes included overall survival (time from histological diagnosis to death from any cause or administrative censoring), surgical resectability, and treatment modality (surgery, chemoradiotherapy, or combined approaches).

Helicobacter pylori status was recorded as positive, negative, or not reported according to the pathology or clinical records available at each institution. Quantitative assessment of bacterial density, colonisation burden, or histological grading was not consistently available and was therefore not included in the analysis.

For the purposes of this study, palliative management was operationally defined as documentation of non-curative treatment intent, including referral to palliative care services, best supportive care, symptom-directed treatment, or oncological management in the setting of unresectable, metastatic, or medically inoperable disease.

Adjustment covariates included age, sex, comorbidities, performance status, histological subtype, and clinical TNM stage when available.

### Data sources and collection procedures

Data were obtained from institutional pathology and endoscopy databases, electronic medical records, and surgical registries. At each centre, a designated investigator performed data extraction using a standardised multicentre case report form (CRF), harmonised across institutions. Data were subsequently consolidated into a unified central database.

Classification as PEGC was confirmed through cross-verification of the prior “negative” endoscopy reports and their associated histology, matched against the confirmatory pathological report of gastric cancer. Tumour location was determined based on endoscopic, surgical, and pathological reports, with pathological assessment taking precedence in cases of discrepancy. Vital status was ascertained from the most recent clinical record and institutional death certificates.

### Strategies to minimise bias

To reduce exposure misclassification, a standardised operational definition of PEGC (6–36 month window) was applied, and case classification was validated by an interdisciplinary clinico-pathological committee comprising gastroenterologists, pathologists, and oncological surgeons.

To mitigate information bias, a single CRF was employed, data extractors underwent standardised training, and a 10% inter-institutional audit of records was conducted. Additionally, central pathological re-review was performed by a reference pathologist.

Given the multicentre design, systematic inter-hospital differences were controlled through centre adjustment in statistical models and estimation of hospital-clustered robust standard errors.

### Statistical analysis

Statistical analyses were performed using SPSS version 25 and R version 4.3.2. Categorical variables were summarised as absolute and relative frequencies, whilst continuous variables were described using mean ± standard deviation (SD) or median (interquartile range, IQR), as appropriate according to distribution.

Between-group comparisons were conducted using the χ² test or Fisher’s exact test for categorical variables, and Student’s t-test or the Mann–Whitney U test for continuous variables.

Overall survival was estimated using Kaplan–Meier curves and compared using the log-rank test. Multivariable logistic regression was used to identify factors associated with PEGC. Differences in survival were evaluated using Cox proportional hazards models adjusted for age, sex, tumour stage, and histological subtype.

All models incorporated hospital-clustered robust variance estimators. Sensitivity analyses were conducted using mixed-effects models with centre as a random effect and alternative exposure windows (12–24 months). Continuous variables were retained in their original form whenever possible; when categorised, clinically relevant cut-off points were reported. A two-sided p value <0.05 was considered statistically significant.

### Quality control and validation

The central analytical database incorporated automated validation rules, change tracking, and systematic review of inconsistencies. A 10% cross-institutional record review was performed, along with central pathological re-evaluation and committee adjudication to confirm PEGC classification.

### Ethical considerations

The study protocol was approved by the institutional research ethics committees of all participating centres and conducted in accordance with the Declaration of Helsinki (15) and Colombian national regulations (Resolution 8430 of 1993) (16). Given the retrospective design and use of anonymised data, informed consent was waived. The consolidated database was stored on secure institutional servers with restricted access limited to authorised investigators.

## Results

### Cohort characteristics

599 patients with histologically confirmed gastric neoplasms were included. The mean age of the cohort was 66.0 ± 12.3 years, and 58.1% were female. Seventy-one patients fulfilled criteria for post-endoscopy gastric cancer (PEGC), corresponding to a frequency of 11.9% (95% CI: 9.5–14.7), whilst 528 patients (88.1%) had no documented upper gastrointestinal endoscopy within the 36 months preceding diagnosis.

No statistically significant differences were observed between groups in mean age (66.6 ± 10.2 vs 66.0 ± 12.6 years; p=0.610) or sex distribution (54.9% vs 58.5% female; p=0.654), indicating comparable baseline demographic characteristics (**Table 1**).

**Table 1:** Baseline characteristics by group.

Variable	PEGC (n = 71)	No prior EGD (n = 528)	Total (n = 599)	P value
Age, mean ± SD (years)	66.6 ± 10.2	66.0 ± 12.6	66.0 ± 12.3	0.61
Female, n (%)	39 (54.9)	309 (58.5)	348 (58.1)	0.654
History of other tumours, n (%)	1 (1.4)	7 (1.3)	8 (1.3)	1.000
*H. pylori* positive, n (%)	50 (70.4)	352 (66.7)	402 (67.1)	
*H. pylori* negative, n (%)	21 (29.6)	174 (33.0)	195 (32.6)	
*H. pylori* not reported, n (%)	0 (0.0)	2 (0.4)	2 (0.3)	χ² (3 categories), p = 0.734

Data are presented as mean ± SD or n (%).Abbreviations: EGD, esophagogastroduodenoscopy; SD, standard deviation; PEGC, post-endoscopy gastric cancer.

Comparisons were performed using Student’s t-test for continuous variables and the χ² test or Fisher’s exact test for categorical variables. Percentages were calculated using the denominator of each column; totals may not equal 100% due to rounding.

### *Helicobacter pylori* infection and quality of prior endoscopy

Overall *Helicobacter pylori* positivity was high in the entire cohort (67.1%). The proportion was numerically higher in the PEGC group (70.4%) compared with patients without prior endoscopy (66.7%), although this difference was not statistically significant (p=0.734). No relevant differences were observed in the distribution of negative or unreported results between groups. This variable reflected the presence or absence of infection only. Information regarding bacterial density, colonisation burden, or histological grading was not systematically available across participating centres.

Amongst patients classified as PEGC, documentation of systematic gastric mapping according to the Sydney protocol was identified in 21 cases. Although this finding suggests that guideline-based assessment was performed in a subset of patients, the retrospective design and incomplete availability of procedural details precluded comprehensive evaluation of adherence to all recommended quality indicators (**Table 1**).

### Anatomical tumour location

In the overall cohort, the most frequent tumour locations were the antrum (30.7%), gastric body (28.0%), and combined body–antrum involvement (17.7%).

When stratified by exposure group, a higher proportion of antral tumours was observed amongst patients with PEGC (36.6%; 95% CI: 25.4–48.9) compared with those without prior endoscopy (29.9%; 95% CI: 25.9–34.1). Conversely, tumours located in the gastric body were more frequent in patients without prior endoscopy (28.8% vs 22.5%).

The global comparison of anatomical tumour distribution differed significantly between groups (Pearson’s χ² = 14.21, df = 6, p = 0.028). However, no individual anatomical location demonstrated a statistically significant difference when evaluated separately. Therefore, these findings should be interpreted as differences in overall distribution patterns rather than evidence of a specific anatomical predilection associated with PEGC (**Table 2**).

**Table 2:** Tumour location by exposure group (PEGC vs No Prior EGD).

Tumour location	PEGC (n = 71)	No prior EGD (n = 528)	Total (n = 599)	P value (site comparison)
Antrum	27 (38.0%; 95% CI 26.8–50.3)	158 (29.9%; 95% CI 25.9–34.1)	185 (30.9%)	0.172
Antrum and incisura	5 (7.0%; 95% CI 2.3–15.7)	67 (12.7%; 95% CI 10.0–15.9)	72 (12.0%)	0.181
Pyloric antrum	5 (7.0%)	26 (4.9%)	31 (5.2%)	0.354
Body	16 (22.5%; 95% CI 13.7–34.0)	152 (28.8%; 95% CI 25.0–33.0)	168 (28.0%)	0.264
Body and antrum	15 (21.1%)	91 (17.2%)	106 (17.7%)	0.41
Incisura	3 (4.2%)	28 (5.3%)	31 (5.2%)	0.707
Gastroesophageal junction	0 (0.0%)	6 (1.1%)	6 (1.0%)	0.408*

The global comparison of anatomical tumour distribution differed significantly between groups (Pearson’s χ² = 14.21, df = 6, p = 0.028). However, no individual anatomical location demonstrated a statistically significant difference when evaluated separately.

95% confidence intervals for proportions were calculated using the exact binomial method (Clopper–Pearson).

Fisher’s exact test was used when the expected cell count was <5.

PEGC, post-endoscopy gastric cancer; EGD, esophagogastroduodenoscopy.

### Findings at prior endoscopy amongst PEGC cases

Amongst patients subsequently classified as PEGC, the most frequently documented findings at prior endoscopy were intestinal metaplasia (35.2%), gastric atrophy (22.5%), and erosions (12.7%). These findings indicate that a substantial proportion of cases later diagnosed as PEGC initially presented with premalignant lesions or subtle mucosal abnormalities, without endoscopic evidence of overt malignancy at the time of the initial examination.

### Hospital outcomes

Clinically and statistically significant differences were observed in hospital outcomes between groups. Patients with PEGC exhibited a markedly higher proportion of palliative management (23.9% vs 2.8%), increased in-hospital mortality (16.9% vs 7.0%), and a substantially lower likelihood of discharge to outpatient follow-up (11.3% vs 74.4%) compared with patients without prior endoscopy (global χ² p<0.001) (**Table 3**).

**Table 3:** Outcomes at hospital discharge by group (PEGC vs No Prior EGD).

Outcome at discharge	PEGC (n = 71)	No prior EGD (n = 528)	Total (n = 599)	OR (95% CI) PEGC vs No Prior EGD	P value
Outpatient discharge	8 (11.3%; 95% CI 5.0–20.9)	393 (74.4%; 95% CI 70.4–78.1)	401 (66.9%)	0.044 (0.020–0.093)	<0.001
Oncology (discharge/follow-up)	28 (39.4%)	58 (11.0%)	86 (14.4%)	5.49 (3.17–9.49)	<0.001
Palliative management	17 (23.9%; 95% CI 14.8–35.3)	15 (2.8%; 95% CI 1.6–4.6)	32 (5.3%)	10.77 (5.09–22.77)	<0.001
In-hospital death	12 (16.9%; 95% CI 9.0–27.7)	37 (7.0%; 95% CI 5.0–9.5)	49 (8.2%)	2.70 (1.33–5.46)	0.004
Surgery/surgical management	4 (5.6%)	14 (2.7%)	18 (3.0%)	2.14 (0.67–6.39)	0.18
Remission	1 (1.4%)	2 (0.4%)	3 (0.5%)	3.77 (0.33–42.4)	0.357*
Discharge against medical advice	0 (0.0%)	4 (0.8%)	4 (0.7%)	—	0.999*
Lost to follow-up	0 (0.0%)	5 (0.9%)	5 (0.8%)	—	0.999*
Other/Not reported	1 (1.4%)	0 (0.0%)	1 (0.2%)	—	0.123*

Global test of distribution (all categories): Pearson’s χ² = 128.7; df = 8; p< 0.001.

95% CIs for proportions were calculated using the exact binomial method.

Odds ratios (ORs) and 95% CIs were calculated using the Woolf logarithmic method.

Fisher’s exact test was used when expected cell counts were <5.

PEGC, post-endoscopy gastric cancer; EGD, esophagogastroduodenoscopy.

A higher proportion of palliative care management and in-hospital mortality was observed amongst PEGC cases, with OR >10 for palliative care and OR ≈2.7 for mortality.

In bivariate analyses, PEGC was associated with a substantially increased risk of palliative management (OR 10.77; 95% CI: 5.09–22.77; absolute risk difference +21.1 percentage points; p<0.001) and in-hospital mortality (OR 2.70; 95% CI: 1.33–5.46; absolute risk difference +9.9 percentage points; p=0.004). Conversely, PEGC was strongly associated with a reduced probability of discharge to outpatient care (OR 0.044; 95% CI: 0.020–0.093; absolute risk difference −63.2 percentage points; p<0.001) (**Table 4**).

**Table 4:** Relative and absolute effects on selected outcomes.

Outcome	PEGC	No prior EGD	OR (95% CI)	RD, pp (95% CI)	P value
Palliative care	17/71 (23.9%)	15/528 (2.8%)	10.77 (5.09–22.77)	+21.1 (11.1 to 31.1)	<0.001
In-hospital death	12/71 (16.9%)	37/528 (7.0%)	2.70 (1.33–5.46)	+9.9 (0.9 to 18.9)	0.004
Outpatient discharge	8/71 (11.3%)	393/528 (74.4%)	0.044 (0.020–0.093)	−63.2 (−71.4 to −54.9)	<0.001

OR, odds ratio; RD, risk difference expressed in percentage points (pp). P values were calculated using the χ² test (2×2). 95% CIs were calculated using the logarithmic approximation for OR and the Wald method for RD.

## Discussion

In this multicentre cohort of 599 patients treated at tertiary university referral hospitals in Colombia, post-endoscopy gastric cancer (PEGC) accounted for 11.9% of all gastric cancer diagnoses, a proportion situated at the upper boundary of estimates reported internationally. This finding is particularly noteworthy given the absence of structured endoscopic screening programmes in our setting and suggests that the magnitude of PEGC in Latin America may be comparable to, or even exceed, that observed in regions with organised early detection strategies (6, 7). Collectively, these results indicate that PEGC is a clinically relevant phenomenon with important implications for the quality of upper gastrointestinal endoscopy in the region. The high prevalence of Helicobacter pylori infection observed in our cohort further reinforces the role of microbial-host interactions in gastric carcinogenesis and highlights the importance of integrating infection control strategies with improvements in endoscopic quality.

Reported PEGC rates in Asia and Europe demonstrate substantial variability depending on baseline disease incidence, population characteristics, and the presence of screening programmes. In high-incidence Asian countries with established population-based strategies, such as Japan and South Korea, PEGC proportions range between 5% and 11% in high-risk cohorts undergoing intensive surveillance (17–19). In Europe, where systematic endoscopic screening is not widely implemented, overall frequencies tend to be lower in the general population, although comparable rates have been described in selected surveillance cohorts (20, 21). Within this comparative framework, the frequency observed in our Colombian cohort is striking because it mirrors figures reported in structured surveillance settings despite the absence of organised screening policies. This observation suggests that, in high-burden regions without formal screening infrastructure, PEGC may be more closely related to endoscopic quality than to procedural access alone.

Several patient-, tumour-, and procedure-related factors have been consistently associated with an increased risk of PEGC. Amongst patient-related factors, chronic Helicobacter pylori infection, extensive gastric atrophy, intestinal metaplasia, advanced age, and a family history of gastric cancer have been identified as important determinants of gastric carcinogenesis and may increase the likelihood of lesions being overlooked during routine endoscopic evaluation (6, 7, 22–25). Furthermore, diffuse-type gastric cancer represents a particular diagnostic challenge because of its infiltrative growth pattern, limited mucosal disruption, and tendency to present with subtle endoscopic findings that may be difficult to recognise during conventional examination (6, 7). Tumour-related characteristics also contribute substantially to missed diagnoses, particularly small lesions, superficially depressed or flat neoplasms, isochromatic lesions with minimal contrast relative to the surrounding mucosa, and lesions located in anatomically challenging areas such as the incisura angularis and proximal gastric body, which have repeatedly been identified as frequent sites of diagnostic omission (7, 8, 10, 22). In addition, procedure-related factors play a central role in PEGC development, including inadequate inspection time, incomplete mucosal visualisation due to suboptimal cleansing, failure to obtain systematic biopsies in patients with premalignant conditions, and lack of appropriate endoscopic surveillance following the identification of gastric atrophy or intestinal metaplasia (7, 8, 10, 22–25).

A key finding of our study was the high prevalence of mucosal abnormalities documented at prior endoscopy amongst PEGC cases, particularly intestinal metaplasia (35.2%) and gastric atrophy (22.5%). This pattern is consistent with international evidence indicating that most post-endoscopy gastric cancers do not represent rapidly progressive neoplasms but rather pre-existing lesions that were overlooked or underestimated during the initial examination (6–8). Perceptual errors remain amongst the most frequently implicated mechanisms, especially in small lesions or those arising on a background of chronic inflammatory or metaplastic mucosa, and may account for a substantial proportion of missed cancers in previous series (8). The coexistence of documented premalignant lesions at prior endoscopy suggests that missed lesions or incomplete surveillance may have contributed to a proportion of PEGC cases. However, the absence of formal root-cause adjudication precludes definitive attribution of individual cases to specific mechanisms.

Although PEGC is frequently regarded as a marker of missed lesions and deficiencies in endoscopic quality, a negative endoscopic examination should not be interpreted as complete protection against future gastric cancer development. Whilst most PEGC cases are believed to result from lesions that were overlooked, incompletely characterised, or inadequately surveilled during a previous examination, a minority may represent biologically aggressive neoplasms that evolve within a relatively short period despite an apparently normal endoscopic assessment. This phenomenon appears particularly relevant for diffuse-type gastric cancer, which often exhibits an infiltrative growth pattern, subtle mucosal changes, and limited endoscopic conspicuity, thereby increasing the likelihood of interval cancer development even when conventional quality standards are met (6–8).

Technical quality of endoscopy emerges as a central determinant of PEGC. Studies from Asia and Europe consistently demonstrate that insufficient inspection time, incomplete mucosal visualisation, suboptimal cleansing, and lack of systematic examination are associated with increased risk of missed early gastric lesions (7, 8, 10, 22). In particular, observation times shorter than three minutes have been associated with a higher risk of advanced post-endoscopy gastric cancer, whereas prolonged inspection and adherence to structured examination protocols appear protective (10, 22). Documentation of Sydney protocol sampling was identified in fewer than one-third of PEGC cases. However, because procedural details were incompletely available, neither adherence nor appropriateness of protocol implementation could be formally assessed. Consequently, the present data should not be interpreted as evidence of inadequate protocol compliance but rather as an indication of limited availability of documentation regarding systematic gastric mapping in prior examinations.

Beyond conventional quality indicators, advances in image-enhanced endoscopy and structured endoscopic assessment may represent important opportunities to reduce the burden of PEGC (23–25). Improved mucosal visualisation, systematic examination protocols, and emerging computer-assisted detection systems have shown promise in facilitating recognition of subtle neoplastic and premalignant lesions that may otherwise remain undetected. Given the high frequency of PEGC observed in our cohort, future prospective studies in Latin America should evaluate whether incorporation of these approaches into routine clinical practice improves lesion detection, optimises surveillance strategies, and ultimately reduces PEGC occurrence in high-incidence populations.

Although the overall distribution of tumour locations differed between groups, no individual anatomical site demonstrated a statistically significant association with PEGC. Therefore, the clinical significance of the observed distribution pattern remains uncertain and should be interpreted cautiously. This finding contrasts with some international reports, where interval cancers have been more frequently identified in the gastric body and incisura angularis, recognised endoscopic blind spots (7, 8). The pattern observed in our cohort could reflect epidemiological and pathogenetic particularities of the studied population, characterised by high Helicobacter pylori prevalence and chronic antral gastritis, both of which may obscure subtle mucosal abnormalities in this region. These findings suggest that the anatomical distribution of missed lesions may vary according to local epidemiology and endoscopic practice patterns.

Although no individual anatomical location demonstrated a statistically significant association with PEGC, the numerically higher proportion of antral lesions observed amongst PEGC cases raises the possibility that certain antral neoplasms may be more difficult to recognise during routine endoscopic examination. Chronic inflammation, mucosal erythema, atrophic changes, and intestinal metaplasia are frequently concentrated in the distal stomach and may obscure subtle neoplastic lesions, potentially contributing to diagnostic oversight. This hypothesis should be interpreted cautiously because the present study was not specifically designed to evaluate location-specific miss rates, but it warrants further investigation in prospective studies incorporating detailed endoscopic image review and formal root-cause adjudication.

Beyond its frequency, PEGC in our cohort was associated with markedly unfavourable hospital outcomes, including higher rates of palliative management, increased in-hospital mortality, and substantially reduced likelihood of discharge to outpatient care. These findings are concordant with evidence from Asian cohorts, where gastric cancer diagnosed following a negative endoscopy is often detected at more advanced stages, with lower curative resection rates and poorer clinical outcomes (26–28). Although cancer stage at diagnosis was not consistently available across participating centres, the substantially higher rates of palliative management and mortality observed amongst PEGC patients strongly suggest that many of these tumours were diagnosed at an advanced clinical stage. Delayed detection limits the feasibility of curative approaches, including endoscopic resection and potentially curative surgery, and frequently necessitates more aggressive or palliative treatment strategies, thereby increasing both patient morbidity and healthcare system burden.

Taken together, our findings indicate that PEGC occurs at a frequency comparable to that reported in countries with organised screening programmes but remains associated with substantially worse clinical outcomes. This contrast suggests that, in high-incidence settings without structured screening programmes, PEGC may reflect, at least in part, potentially modifiable factors related to endoscopic practice and surveillance. From a health systems perspective, these results support prioritising systematic quality-improvement interventions, including protocol standardisation, performance auditing, structured surveillance of premalignant gastric lesions, and routine monitoring of PEGC rates as a quality indicator.

This study has several methodological strengths, including its multicentre design, sizeable cohort, and use of a standardised operational definition of PEGC aligned with international literature, thereby enhancing external comparability. Cross-verification of endoscopic and pathological records reduced exposure misclassification and strengthened internal validity. Nevertheless, certain limitations should be acknowledged. The retrospective design precludes causal inference and limited detailed assessment of important technical variables such as inspection time, mucosal preparation quality, and utilisation of image-enhancement technologies, which were not consistently documented. Cancer stage at diagnosis was not uniformly available and therefore could not be comprehensively analysed. Furthermore, the inability to directly review prior endoscopic images restricted detailed evaluation of specific omission mechanisms. The inclusion of distinct gastric neoplasms, including adenocarcinoma, lymphoma, gastrointestinal stromal tumours, and neuroendocrine neoplasms, represents an additional limitation. These entities differ substantially in biological behaviour, endoscopic appearance, growth kinetics, and clinical outcomes. Because histology-specific analyses were not available, the present results should primarily be interpreted as describing post-endoscopy gastric neoplasia rather than exclusively post-endoscopy gastric adenocarcinoma. An important limitation is that a formal root-cause analysis according to established PEGC or post-endoscopy upper gastrointestinal cancer classifications was not feasible because detailed review of original endoscopic images, procedural videos, and complete pathology documentation was not consistently available. Consequently, individual PEGC cases could not be classified as missed lesions, incomplete examinations, inadequate surveillance, sampling errors, or true interval cancers. Future studies should incorporate standardised root-cause adjudication frameworks to better identify actionable mechanisms underlying PEGC occurrence. Despite these limitations, this study represents, to our knowledge, the first multicentre study specifically evaluating PEGC in Latin America. Future prospective multinational studies involving larger cohorts across the region are warranted to better define PEGC incidence, risk factors, stage distribution, endoscopic quality indicators, and long-term oncological outcomes.

## Conclusions

In this multicentre Colombian cohort, post-endoscopy gastric cancer (PEGC) was frequent and occurred at a proportion comparable to that reported in regions with structured screening programmes, despite the absence of organised endoscopic screening. Patients with PEGC experienced significantly worse clinical outcomes, including a higher need for palliative management and increased in-hospital mortality, underscoring the clinical impact of delayed diagnosis and suggesting missed diagnostic opportunities.

Our findings suggest that a proportion of PEGC cases may be related to potentially modifiable factors in endoscopic practice. However, prospective studies incorporating formal root-cause adjudication are needed before specific mechanisms can be definitively established. In this context, we propose incorporating the PEGC rate as a key institutional performance indicator in referral centres, complemented by the establishment of minimum endoscopic quality standards. These should include adequate inspection times, systematic examination of the gastric mucosa, structured photographic documentation, and adherence to the Sydney protocol.

Furthermore, implementation of institutional endoscopic quality checklists and periodic performance audits may represent pragmatic and potentially cost-effective strategies to reduce missed premalignant lesions, enhance early detection, and ultimately decrease the clinical burden of gastric cancer in high-incidence regions.

## Data Availability

The original contributions presented in the study are included in the article/supplementary material. Further inquiries can be directed to the corresponding author.
